# Sámi youth health, the role of climate change, and unique health-seeking behaviour

**DOI:** 10.1080/22423982.2018.1454785

**Published:** 2018-03-22

**Authors:** Emilie Kowalczewski, Joern Klein

**Affiliations:** a Faculty of Health, Maastricht University, Maastricht, The Netherlands; b Faculty of Health and Social Sciences, Department of Nursing and Health Sciences, University College Southeast Norway, Kongsberg, Norway

**Keywords:** Health and well-being, climate change, Sami youth, reindeer herding, “Norwegianization”

## Abstract

The goal of this cross-sectional qualitative study was to assess the impact of climate change on Sámi youth health, health care access, and health-seeking behaviour. Indigenous research methodology served as the basis of the investigation which utilised focus groups of youths and one-on-one interviews of adult community leaders using a semi-structured, open-ended questions. The results of the focus groups and interviews were then analysed to identify trends. We found that Sámi youth mostly associate the implications of climate change to their culture andcultural practices rather than the historical influence the environment had on Sámi health. They also take part in unique health-seeking behaviour by utilising both traditional and Western medicine simultaneously but without interaction due to social and structural factors. Our findings suggest that the health of Sámi teens is not tied to the environment directly, but through cultural activities.

## Background

Sámi identity is strongly tied to reindeer herding. Currently, only 10% of the Sámi population actively herd reindeer, but it is so integral to Sámi identity that Sámi easily identify and characterise themselves as either reindeer herder or not ^[1]^. Reindeer herding can be physically dangerous due to the long distances traversed on rough terrain and the risks associated with handling reindeer, and may be linked to mental health problems [2–4]. Altered ice stability can also increase physical and economic stress on reindeer herders as the terrain is no longer capable of sustaining the movement of reindeer herds to new pastures [1,5]. The accelerated movement of industries into the Arctic, together with the accompanying increase of infrastructure and decreased snow cover from climate change, has a negative impact on reindeer herders by reducing available pastures for their animals [5,6].

Sami health needs to contextualised with the historical “Norwegianization” of the Sámi in Norway which targeted children in the form of boarding schools from the 1840s to the 1950s, to fully understand the current impact of environmental and cultural changes in Sámi populations [7]. The ability to internalise Sámi-associated cultural support versus cultural shame has been found to have an impact on Sámi youth health and cultural continuity [8]. In order to countervail this, the Norwegian government passed legislation in the 1950s that makes it impossible to ethnically identify as anything other than Norwegian [7] . Therefore, it is not possible to get population statistics or health statistics on ethnic groups that identify as Sámi in anyway beyond the number of Sámi individuals associated with the Sámi parliament. However, Norwegian Arctic regions that are highly populated with Sámi individuals have higher unemployment rates and reported more cases of sexually transmitted diseases, higher smoking rates, lower education rates, lower income, and higher illiteracy rates than the Norwegian average [9–11].

The impact of climate change could particularly impact Sámi youth health as they become the next generation of reindeer herders and members of the surrounding Sámi community which, due to geography, is more likely to be strongly impacted by climate change [5]. The Sámi Arctic youth are also in an area with lower social determinants of health which could potentially include a previously unexplored link with the environment and climate change which has been recorded to be very important for the Sámi historically [12].

## Methods

Sámi high school students (16–19 years) from Kautokeino were invited to share their perceptions and views of climate change, its interaction with their well-being, and coping strategies that they have available to them using open-ended, semi-structured interview questions in focus groups (one group of three reindeer-herding teens and two groups of three non-herding teens).

Nine different community leaders from diverse backgrounds were interviewed one-on-one using similar open-ended questions to provide a holistic perspective and context for the youth’s responses. Transcripts from the focus groups and interviews were then analysed for trends in subject matter and opinion. All data, analysis, and conclusions were made available to the community as per indigenous research practices [13].

## Results

The results of this study indicated that climate change has an impact on Sámi health, notably, that those individuals involved in reindeer herding, or hoping to be involved in the future, are particularly susceptible to be negatively impacted by climate change by increased safety issues (such as less stable ice and snow routes) or through anxiety. Anxiety tied to climate change was quite commonly described and tended to be related to cultural continuity, especially the feasibility and opportunity for economic development of reindeer herding in the future.

“The constant worry is also about the weather, the animals, and the windmills [and how it affects] the family that cares for reindeer…. It’s a lot of stress… I mentioned earlier that our whole life is [centered] around the reindeer and how everything that happens affects the reindeer. From people walking in the mountain to expropriation to weather to cabins being built to the mining industry, everything. It’s always about how does this affect the reindeers.” (edited for clarity)

When asked about the health-seeking process which they may undergo as a theoretical exercise, all the participants shared similar responses. After exhausting any individual-coping strategies, they would first seek help and support from family. Friends were also mentioned; however, many close friends were family members as well (i.e. cousins). The respondents mentioned that if the trouble was so severe that family could not handle the issue, they would then resort to seeking professional aid. Rather than seeking medical attention to handle medical events before they became severe, the Sámi participants tended to wait until their medical concerns are severe; seeking medical aid was the final option.

## Discussion

**Figure 1.**
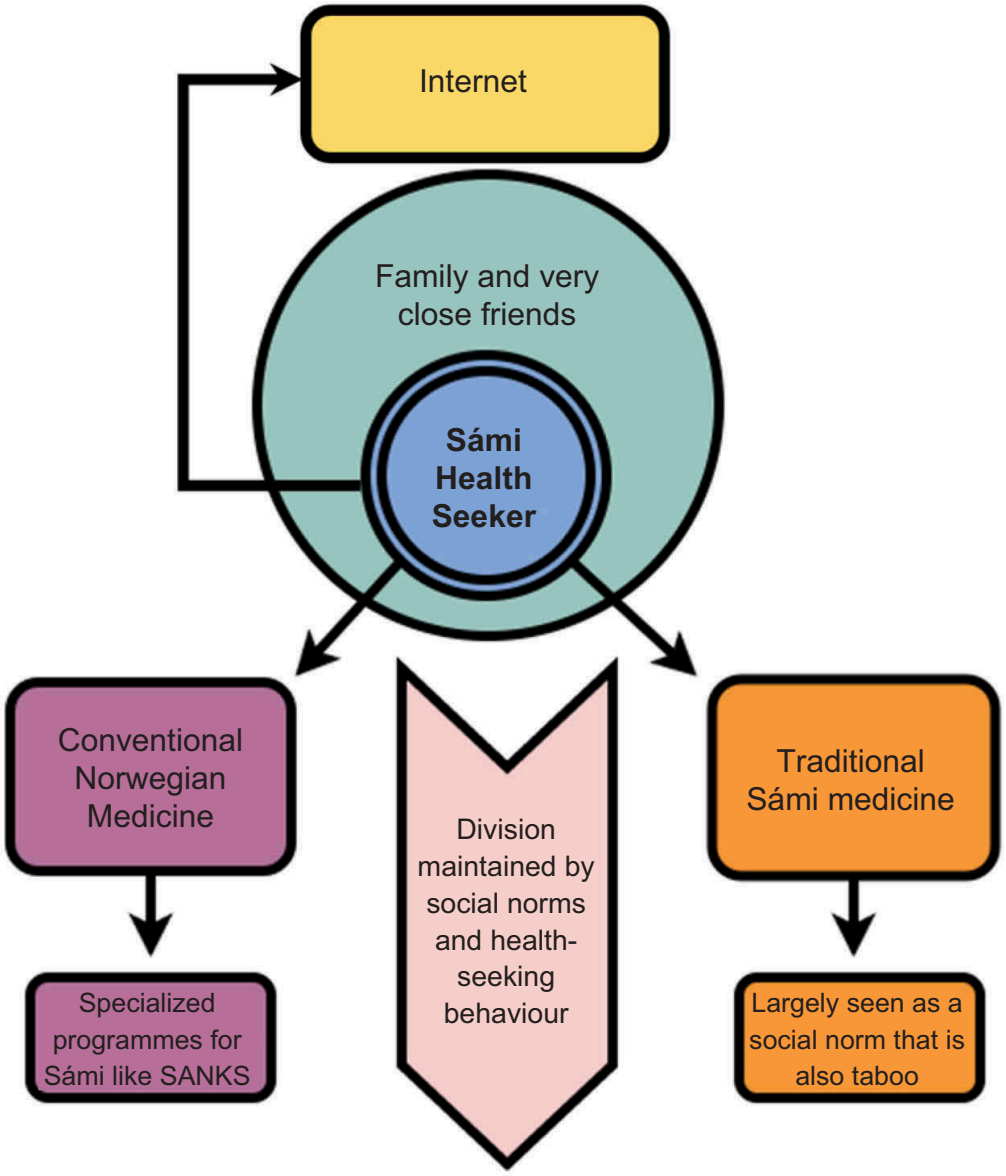
Health-seeking behaviour model with gatekeepers and health-seeking directions and patterns, notably parallel health-seeking behaviour after exhausting individual and social support. The Norwegian and traditional Sámi health systems are separated by social norms and health-seeking behaviours.

Due to social and cultural pressures, Sámi health-seeking behaviour is unique in that it has several gatekeepers within it that keep health-seeking behaviour along several paths (Figure 1). The first one is the individual who must recognise that they require medical attention. The second gatekeeper is family and friends who must accept that the health seeker has an issue which cannot be resolved by their social support network; this was described as a difficult thing to achieve as Sami culture often values the hardiest and most independent individuals the most. In fact, all participants described coping mechanisms which were highly individual like journaling, going out to the reindeer pastures alone, and hiking rather than communal coping mechanisms like talking to friends and family or group exercise. Finally, the medical system has its own set of gatekeepers. Within the Western medical framework, the gatekeeper is the medical professional due to necessary referrals to access specialised programmes. The traditional medical system has a non-specific gatekeeper which is stigma for traditional medical beliefs. Almost all respondents made it clear that they did not allow these branches to intersect and the health professionals interviewed stated that often their patients deny their use of traditional medical practices due to stigma. The youth also described the influence of the internet as a source of greater social support which they relied on to express themselves and to seek advice.

Interestingly, many women and girls who participated in the study identified that they worried about other’s health and health-seeking behaviour as much as their own. This suggests that the current gatekeeper system used by the Sámi puts pressure put on individuals, often women, within the family to be aware and conscious of the health status of other family members.

Unlike the impacts of globalisation and climate change on culture, Sámi youth had difficulty identifying the impacts of global forces on their health. This suggests that the Sámi no longer define their health in the context of the environment like they did around 100 years ago [12]. Instead, they seem to have adapted to the Norwegian health system and health-based beliefs. Therefore, what is and what is not good health is no longer dictated by Sámi culture, but rather, the concept of health has been adapted from Norwegian society while certain aspects of Sámi healing culture have remained as a form of expression of traditional Sámi culture. As a result, culture is the main vehicle by which climate change impacts the Sámi.

This may explain why anxiety about the future was a consistent way in which the influence of climate change was described by Sámi participants as climate change does not only influence the environment for the Sámi. Rather, it directly affects culture in a population that has had its identity and culture previously targeted and attacked – climate change in the new “Norwegianization” and can potentially erase swathes of Sámi culture. The worry associated with climate change’s effect on the Sami may be contributing to the growing mental health concerns in the Arctic [1–3]. Considering that climate change is a current problem in the North, it will be beneficial to create and promote cultural programmes and practices as an integral part of health policy and for the improvement of Sámi health and well-being in the Arctic. Future research should determine if the value of culturally based health programmes such as reintegrating a health professional into Sámi siidas or providing one-on-one therapy outdoors/on the tundra would be beneficial to the community. The role of women in Sámi health in this time of transition and change is also worth further investigation.
